# Translation, Cross-Cultural Adaptation and Validation of the Lymphedema Quality of Life Questionnaire (LYMQOL) in German-Speaking Patients with Upper Limb Lymphedema

**DOI:** 10.3390/healthcare12181881

**Published:** 2024-09-20

**Authors:** Torsten Schulz, Mary Lee Warg, Simon Heister, Kristin Lidzba, Günce Ciklatekerlio, Yasmin Molter, Stefan Langer, Rima Nuwayhid

**Affiliations:** Department of Orthopaedic, Trauma and Plastic Surgery, University Hospital Leipzig, 04103 Leipzig, Germany; mary.warg@medizin.uni-leipzig.de (M.L.W.); simon.heister@medizin.uni-leipzig.de (S.H.); kristin.lidzba@medizin.uni-leipzig.de (K.L.); guence.ciklatekerlio@medizin.uni-leipzig.de (G.C.); yasmin.molter@medizin.uni-leipzig.de (Y.M.); stefan.langer@medizin.uni-leipzig.de (S.L.); rima.nuwayhid@medizin.uni-leipzig.de (R.N.)

**Keywords:** lymphedema, LYMQOL, upper limb lymphedema, quality of life, physiotherapy, decongestive therapy, lymphatic surgery

## Abstract

**Objectives:** The LYMQOL is the most frequently translated, validated, objective tool for assessing Health-Related Quality of Life (HRQoL) in individuals with upper limb lymphedema (ULL). There have been adaptations and validations in a number of different countries. While a German version of LYMQOL Leg for lower limb lymphedema is available, a validated translation of LYMQOL Arm to German is lacking. We aimed to perform a cross-cultural adaption and translation according to ISPOR principles. **Methods:** Patients suffering from ULL from Germany, Austria, and Switzerland (n = 52) were questioned twice using the translated LYMQOL Arm, SF-36, and an evaluation questionnaire. The assessment of the content validity and face validity proved this version of LYMQOL Arm to be acceptable for interviewing German-speaking patients with ULL in Germany. **Results:** Comparison of LYMQOL Arm and SF-36 demonstrated good construct validity. Test–retest reliability was moderate to good (intraclass correlation coefficients 0.66–0.73). Cronbach’s alpha values varied between 0.79 and 0.89 in both interviews, indicating good internal consistency. Factor analysis revealed a cumulative variance of 59.5% for the four domains of the questionnaire. **Conclusion:** There was no significant association between lymphedema stage and LYMQOL Arm score. This trial tested the appropriateness of the German version of the LYMQOL Arm for measuring HRQoL in German-speaking individuals with ULL.

## 1. Introduction

Lymphedema is a chronic disease and a manifestation of pathological conditions of the interstitial lymphatic system, resulting in an increase in protein-containing fluid in the interstitium of the affected regions [1]. Primary lymphedema due to the abnormal development of the lymphatic system is rare [2]. Internationally, the most frequent etiology of lymphedema is the illness lymphatic filariasis, mainly caused by the parasitic nematode *Wucheria bancrofti* [3]. In high-income countries, lymphedema is mostly secondary to malignant diseases and their treatment, e.g., axilla dissection and radiotherapy following breast cancer, melanoma, and other cancers [2,3,4,5,6]. Other possible causes are infections, trauma, obesity, and venous diseases [2].

Several risk factors for breast cancer-related lymphedema (BCRL) have been identified, and their acknowledgment in therapy strategies, like sentinel-lymph-node-based management, has reduced the incidence of BCRL [7,8]. Simultaneously, advancements in non-invasive and surgical treatments prolong cancer patients’ lifespan and thus their time of affliction with possible side effects like BCRL [7]. The most common form of cancer in women is breast cancer. One in five breast cancer survivors will develop ULL [4,9].

The swelling of the arm followed by inflammation and fibrosis abet infections, can be painful and progressively impair limb function, spine, and general posture, and can, consequently, affect activities of daily life [10,11,12]. ULL negatively affects physical, psychological, and social well-being, resulting in an impaired quality of life (QoL) [8,11,13,14,15]. Patients themselves best assess their symptoms, constraints, and most of all their individual QoL without observer bias; thus, patient-reported outcome measures (PROMs) are valuable tools to reflect the patient’s perspective [16]. Several disease-specific PROMs for lymphedema exist and have been assessed; a selection of validated PROMs are listed in Table 1 [17,18,19,20]. Three of these are available in German.

As the reported translation and validation process of the German version of Lymph-ICF was designed poorly, it cannot be considered a robustly validated tool [18,22]. Although the FLQA-L was primarily conceptualized in German almost 20 years ago, it has not been utilized in published studies [33]. The LYMPH-Q Upper Extremity Module has been carefully designed and validated as its German translation [25,26]. Keeley’s LYMQOL is currently the most commonly used disease-specific PROM for lymphedema [20,34]. The LYMQOL Leg questionnaire for lower limb lymphedema (LLL) has recently been translated and validated in a German-speaking cohort [33]. Reflective of the demand for an easily manageable tool to assess QoL in ULL patients, LYMQOL Arm has been adapted into several languages [35,36,37,38,39,40]. Despite its ostensible popularity, a German version is still lacking [41].

The purpose of the present trial was to perform a translation and cross-cultural adaptation of the LYMQOL Arm into German and subsequent validation according to the Principles of Good Practice for the Translation and Cultural Adaptation Process for Patient-Reported Outcomes Measures of the International Society for Pharmacoeconomics and Outcomes Research (ISPOR).

## 2. Materials and Methods

### 2.1. LYMQOL Arm

The LYMQOL Arm, a disease-specific PROM for HRQoL in ULL patients, was developed by Keeley et al. in 2010 [32]. In its modified final version, LYMQOL Arm consists of 28 items arranged into 4 domains, each representing a field of HRQoL: Function (10 items), Appearance and body image (5 items), Symptoms (6 items), and Mood (6 items). Patients rate their individual impairment of HRQoL due to their lymphedema on a Likert-like scale varying from “not at all”, equaling a score of 1, to “a lot”, equaling a score of 4. Consequently, LYMQOL score and HRQoL are inversely proportional, meaning that a higher LYMQOL score signifies greater impairment and thus lower HRQoL.

In order to calculate the total LYMQOL score, the scores from each subscale are added together and then divided by the number of items in that subscale. Only domains with at least half of the questions answered are analyzable; otherwise, they are set at nil. In the domain Function, patients are asked to rate the limitations in their leisure activities and are provided with a text field to list examples. The last item is a numeric rating scale for patients to rate their overall QoL ranging from 0 (poor) to 10 (excellent). This item is not included in the score’s calculation.

The original publication of LYMQOL Arm details modifications made by the authors to their initial questionnaire after further statistical analysis of data retrieved during the validation process. Our initial German version is based on this modified, shorter LYMQOL Arm with one exception: we did not remove the question “Does it affect your relationship with your partner?” (see Section 4). Two items asking the individuals to assess their perceived swelling of each arm on the identical 4-point scale as the following items initiate the survey. This suggestion to make this addition was initially made in the Swedish LYMQOL and was met with positive feedback by patients during the validation of our German version of LYMQOL Leg [33,38].

### 2.2. Short-Form Health Survey (SF-36)

To enable the assessment of the construct and criterion validity of a novel questionnaire, it should be compared to a robustly validated PROM. The SF-36 is an internationally used generic questionnaire used to evaluate global HRQoL with a validated German version [42,43,44,45]. It was utilized in the validation of several PROMs, among them the Italian, Swedish, and Dutch versions of LYMQOL [38,40,46].

Its 36 items are grouped into the physical component summary (PCS) and mental component summary (MCS). To generate the ratings for each of these two domains, weighted subscale scores specific to each language version, in this case German, are added [45]. The final score ranges between 0 and 100. In contrast to LYMQOL, the score is directly proportional to overall QoL, meaning a higher SF-36 score demonstrates higher HRQoL.

### 2.3. Translation Process

The methodology for the translation process was based on the ISPOR principles, with this publication’s senior author as the project manager. Translation, statistics, and validation were performed as described in detail in the publication on the German rendition of LYMQOL Leg [33].

#### 2.3.1. Forward Translation

For forward translations of the English original LYMQOL Arm to German, six clinicians were chosen based on their routine involvement in the treatment of ULL patients, mother tongue (all six native German speakers, with two of them being bilingual in German and English), and gender (three female, three male). In addition to these translations, a professional translator was assigned by a translation and interpretation agency to also perform a translation to German. All translators were briefed on the LYMQOL and its objective, as well as the aim of this study. They were instructed to emphasize broad comprehensibility with average patients in mind.

#### 2.3.2. Reconciliation

After generating seven German versions, the project manager and first author reviewed them and reached a consensus on a preliminary German version.

#### 2.3.3. Back Translation

The draft was presented to the contracted translation agency for reverse translation back into English. This was carried out by a professional translator who was a native English speaker. The translator had no prior knowledge of the initial version.

#### 2.3.4. Back Translation Review and Harmonization

The senior author and the first translator met for a harmonization session to align the back translation with the publication by Keeley and colleagues. Additionally, the initial German version was reviewed by the professional translator. In order to ensure the equality of our translation with Keeley’s original work, we agreed on a preliminary German draft.

#### 2.3.5. Cognitive Debriefing

To gain first insights into the pre-final version’s usability from patients’ perspectives, it was handed out to five ULL inpatients. Their experience in filling in the questionnaire was discussed with them in detail.

#### 2.3.6. Review of Cognitive Debriefing Results and Finalization

The project manager used the insights gathered during the cognitive debriefing to complete the finalization of the German version of LYMQOL Arm.

#### 2.3.7. Proof Reading and Editing

The final version underwent proofreading by all forward translators and a doctoral candidate. A professional designer, instructed to create a visually straightforward layout for easy printing in routine clinical contexts, oversaw subsequent final editing and formatting.

### 2.4. Study Population

Patients with ULL willing to participate in the validation process were enrolled upon visits to our clinic and collaborating outpatient practices for Angiology. Patients presenting with prescriptions entailing lymphedema as a diagnosis to physiotherapeutic practices offering decongestive therapy or to medical supply stores specializing in the customization of compression garments were informed of the study using patient information forms. Furthermore, our call for study participation was disseminated at meetings of self-help groups and online via their network. The latter option was provided to include patients from other German-speaking regions and countries as well as mobility-impaired patients.

### 2.5. Statistics

Data analysis was performed in IBM SPSS Statistics, Version 29 (IBM Corporation, Armonk, NY, USA). To characterize the epidemiological traits of the sample, descriptive statistics were used, including mean, mode, median, standard deviation, and standard error. To assess the validity of the German version of the LYMQOL Arm and to ensure its reliability, we performed a number of statistical procedures to assess face, content, and construct validity.

Using Pearson’s correlation coefficient, we juxtaposed the scores calculated for LYMQOL Arm and SF-36. The intraclass correlation coefficient was computed as a two-way random effects model to check the agreement between the measurements of both questionnaires across both interviews. To examine the questionnaire structure and the importance of each item, a factor analysis was conducted. A factor loading of >0.3 and a cumulative variance of >50% were considered significant. To address missing data, multiple imputations were used, generating five imputed datasets. The threshold for statistical significance was established at less than 0.05 for two-tailed tests.

### 2.6. Validation

#### 2.6.1. Content Validation

We designed a brief third questionnaire consisting of six questions structured either dichotomously or in an open format, mirroring the methodology employed by Wedin et al. in their validation of the Swedish LYMQOL [38]. Its principal aim was to ascertain whether ULL patients perceived LYMQOL Arm as user-friendly and whether it effectively addressed their experiences and concerns.

#### 2.6.2. Criterion and Construct Validity

In order to establish construct validity, the LYMQOL subscales were juxtaposed with the SF-36 MCS and PCS scales considered as benchmarks, alongside patients’ self-reported perceptions of limb swelling. Our hypothesis posited that the MCS would exhibit correlation with LYMQOL domains related to Appearance and Mood, while the PCS would correlate with the domains pertaining to Function and Symptoms. We set a threshold for correlation coefficient values at >0.50, indicating moderate to strong correlations.

#### 2.6.3. Test–Retest Reliability

Individuals were instructed to answer the German versions of LYMQOL Arm and SF-36 on two occasions, separated by a one-week interval. Only datasets containing responses for both questionnaires across both sessions were considered for analysis. The relationship observed across the two assessments was assessed using the intraclass correlation coefficient (ICC), with a threshold ICC of greater than 0.70 considered adequate. Further analyses included using the standard error of measurement (SEM), which quantifies the error across measurements, and the smallest real difference (SRD), which identifies the smallest statistically significant difference.

#### 2.6.4. Internal Consistency

Cronbach’s alpha and factor analysis were conducted to assess internal consistency, with values ranging from 0.70 to 0.95 considered acceptable. To establish a reliable model, a total variance of at least 50% in the factor analysis model was deemed acceptable. Relationships between perceived level of limb lymphedema on the 4 grade Likert scale used throughout the questionnaire and LYMQOL domain scores were examined using the Kruskal–Wallis test and Spearman’s rank correlation coefficient.

## 3. Results

### 3.1. Translation Process

Minimal disparities were observed among the seven forward translations retrieved by the translation process described above, potentially owing to the reasonably simplistic language employed in the original rendition. Robust consensus was achieved regarding the utility of the additional indicators and inquiries. Examination of the backward translation revealed a close semblance to the original English rendition. The Supplementary Files contain the formatted final version of the LYMQOL Arm.

### 3.2. Participants

We obtained analyzable datasets for both questionnaires on both occasions from 52 patients; their descriptive characterization is presented in Table 2. Predominantly female, the cohort included three male participants. On average, the participants were 60 years of age, with a mean duration of ULL diagnosis of 12 years. The average body mass index (BMI) stood at 31.4 kg/m^2^, indicating class 1 obesity. The majority of participants had attained secondary education and were retired at the time of the interview. Half of the patients were classified as having lymphedema stage 2 according to the International Society of Lymphology (ISL) classification. All instances of lymphedema were unilateral, evenly distributed between the right and left arms.

The most frequently selected assessments in the LYMQOL Arm questionnaire, as well as the calculated subscales of the SF-36, are depicted in Table 3.

#### 3.2.1. Completeness

Only participants who completed both rounds of interviews with both the LYMQOL Arm and the SF-36 were included in the analysis. The achieved completeness for both questionnaires was satisfactory, with less than 1% unanswered questions. In the LYMQOL Arm questionnaire, 7 questions remained unanswered in the first round of interviews, with an increase to 11 questions in the second round. The number of unanswered questions in the SF-36 questionnaire was 8 in the first interview and 15 in the second.

#### 3.2.2. Face and Content Validity

As described under 2.6.1., we used a brief questionnaire to review the face and content validity of the German LYMQOL Arm. The results are presented in Table 4. In summary, the prevailing sentiment among participants was that the questionnaire was facile to complete, of appropriate length, the questions clear, and no question unnecessary. Slightly more than one-tenth of participants missed the inclusion of certain areas of their life in the questionnaire, in which they experienced the impact of their lymphedema. This was evidenced by a number of patients listing areas like leisure, work, sexuality, and descriptions of the impact of lymphedema and its therapy on their lives.

#### 3.2.3. Construct Validity

Construct validity analysis revealed correlations between LYMQOL Arm and SF-36 varying from low to high. The weakest correlation was found between the LYMQOL Arm domain Function and the SF-36 PCS subscale with Pearson’s correlation coefficient of −0.33, albeit still considered a statistically significant correlation with *p* ≤ 0.01. With Pearson’s correlation coefficient of −0.73, the LYMQOL Arm domain Mood presented a strong correlation with the SF-36 MCS subscale. While the Appearance domain in LYMQOL showed moderate correlation with the SF-36 MCS subscale, this was not statistically significant, in contrast to the other correlations (Table 5).

Patient-reported lymphedema severity did not significantly relate to LYMQOL domain scores. The domains Function, Appearance, and Symptoms showed a slight increase in the average score values. The Mood domain showed an opposite tendency. The global score showed no trend. The Kruskal–Wallis test did not show a significant difference between the stages in any of the domains (Table 6). Spearman’s correlation showed no relationship between the domains and clinical stages (*p* ≥ 0.05). The overall lymphedema staging was unbalanced, with only 12 people classified as stage I and III and 28 patients classified as stage II.

#### 3.2.4. Test–Retest Reliability

To assess reliability, changes in responses given between the initial interview and the second interview were analyzed for both surveys. Consistency was assessed for each domain for each subscale of the LYMQOL Arm (ICC 0.66–0.73) (Table 7). In line with the accepted standards, these findings were classified as moderate [47].

#### 3.2.5. Internal Consistency

Internal consistency was evaluated using Cronbach’s alpha ranging from 0.79 to 0.89 (Table 8) in the first interview and was rated as acceptable for the domain Symptoms and good for every other domain [48]. In the second interview, the internal consistency for the domains Symptoms, Mood, and Function was rated as good, and Appearance was rated as excellent.

A factor analysis was then carried out (Table 9). Each item that showed a loading above 0.3 was marked in bold. The cumulative variance explained was 59.5% for the LYMQOL Arm with four components (Table 9). Therefore, the factor structure in the four domains was found to be adequate.

#### 3.2.6. Floor and Ceiling Effects

Floor effects ranged from 1.9% for Function to 11.5% for Appearance/Symptoms in the first interview (Table 10). In the second interview, floor effects ranged from 1.9% (Function/Symptoms) to 9.6% (Appearance/Mood). Ceiling effects were observed from 1.9% in Symptoms/Mood to 14.3% in Function in the first interview and from 0% in Function to 5.7% in Appearance in the second interview. The effects did not exceed 15%, suggesting adequate questionnaire design [49].

## 4. Discussion

Lymphedema is a progressive disease that results in the collection of fluid in subcutaneous tissue. It affects millions of individuals around the world, leading to significant morbidity and disabilities. Lymphedema places a substantial burden on the QoL and financial stability of patients. Several assessment tools have been designed to evaluate the impact of lymphedema on QoL. To our knowledge, a validated German version of the originally English LYMQOL Arm is lacking. Our version of LYMQOL Arm fulfilled the quality criteria for health status questionnaires according to Terwee et al. [49]. The questionnaire met the requirements for content validity, internal consistency, construct validity, agreement, reliability, floor or ceiling effects, and comprehensibility [49].

Similar to the Swedish translation, we found a weak to moderate correlation across the physical and mental subscales of the LYMQOL and SF-36 questionnaire [38]. Other questionnaires such as EORTC QLQ-C30 or FACT-B+4 also showed weak to moderate correlation in relation to the LYMQOL Arm in the validation of the Turkish version [37]. In three out of four domains, our arm version demonstrated significant correlations with the PCS and MCS domains. Nevertheless, the LYMQOL domain Function did not show a significant correlation with the SF-36 PCS subscale in the first interview (*p* = 0.16), and the domain Appearance demonstrated no correlation in the second interview (*p* = 0.08). These results suggest a rather low construct validity of the LYMQOL Arm. There was a moderate level of agreement in the test–retest correlation analysis, with correlation coefficients of around 0.700 (ICC). An adequate Cronbach’s alpha of between 0.79 and 0.90 was demonstrated for both interviews with regard to the internal consistency of the questionnaire. The factor analysis revealed a cumulative variance of around 60% for four domains, proving a suitable questionnaire structure. The test–retest reliability was found to be moderate, and the internal consistency was good and similar to the results reported in other studies [38]. Every domain of the questionnaire in every interview showed floor or ceiling effects of less than 15%, indicating an appropriate questionnaire design. As in previous works, no relationship could be found between the clinical stage of the lymphedema of the patients and the questionnaire scores.

Currently, 19 PROMs to assess disease-specific HRQoL in lymphedema are available [50]. None of the questionnaires reviewed had sufficient evidence according to the COSMIN framework to support all nine measurement properties. Among the questionnaires for LLL, the LYMQOL Leg provides sufficient face, structure, and construct validity, as well as internal consistency and reliability [50]. Regarding ULL, the Lymph-ICF-UL had adequate content and construct validity and reliability [19,50]. The original Lymph-ICF-UL is a highly recommended tool, particularly for breast cancer-related lymphedema, due to its strong psychometric properties and comprehensive assessment of physical and psychological impacts [19,21]. Its German translation lacks sufficient validation [22]. ULL-27 is a validated questionnaire measuring the effects of ULL on daily activities and emotional well-being [19,28,51]. The ULL-27 appears to be highly specific to lymphedema, effectively distinguishing it from symptoms caused by axillary lymph node dissection alone, but no German version has been published. The LYMPH-Q Upper Extremity Module is a valuable tool for measuring outcomes important to women with upper extremity lymphedema. Designed using a modern psychometric approach, this new PROM is suitable for both research and clinical care and exists in a robustly validated German version [25,26].

With LYMQOL currently being the most commonly used PROM for lymphedema and due to the good results obtained during the validation process of the LYMQOL Leg, we decided to evaluate the LYMQOL Arm as well [33,34,52].

A stated limitation of LYMQOL is its lack of assessment of lymphedema of the torso, genital area, or head and neck [32]. On the other hand, this restriction is not specific to LYMQOL, as none of the aforementioned instruments can assess non-limb lymphedema.

While our cohort consisting of 53 patients is rather small, it aligns with the sample sizes of other translations and validations of LYMQOL [35,36,46].

We consciously chose an equal number of female and male clinical and professional translators to preclude gender-specific influences on language [53]. Despite this effort, our version is not sufficiently validated for male patients, as we were unable to recruit more than three male patients. However, with the vast majority of ULL patients being women after breast cancer and its therapy, there will still be a large enough number of patients it is applicable to.

During the validation process of LYMQOL Arm, particularly the cognitive debriefing, we were reassured about the slight modifications we made to the original. As explained in more detail in our publication of LYMQOL Leg, we reintroduced the question “Does it affect your relationship with your partner?” removed by Keeley et al. to broadly cover the topic of sexual health [33]. We also transferred the subjective scoring of the perceived swelling as introduced by Wedin et al., the fifth check box column for the answer “not applicable”, as well as a table to facilitate calculating and documenting the score for examiners. Perhaps the most significant alteration was extending the overlying question “How much does your swollen arm affect […]?” to “How much do your swollen arm and *its therapy affect* […]?”. This takes the time-consuming, costly, and often uncomfortable nature of conservative lymphedema therapy into account, which was mentioned in the majority of patient’s comments in personal dialog during validation and cognitive debriefing as well as the evaluation questionnaire [54]. Other comments mainly specified activities impacted by lymphedema, which did not require further adaptation of the questionnaire. For example, patients describing limitations during swimming due to their arm lymphedema are covered by the question “How much does you swollen arm impact your leisure activities?”. Similar considerations are described in the original LYMQOL publication [32,33]. In the original publication as well as its translations, LYMQOL is documented to be comprehensible and easy to use, which is a relevant factor when assessing PROMs autonomously filled out by patients [32,38]. This is achieved through simple language and wording the questions in more general terms. Expanding this concise questionnaire with detailed and specific rather than general questions would subvert its quality of broad applicability.

In summary, this clinical trial provides evidence for the feasibility, validity, and reliability of our LYMQOL Arm version in a German-speaking cohort of mainly female participants with ULL. Its validity is reduced compared to LYMQOL Leg.

## 5. Conclusions

We present a translated and cross-culturally adapted German version of LYMQOL Arm. This provides clinicians and researchers with the possibility of assessing German-speaking ULL and LLL patients with an equivalent tool producing directly comparable scores instead of using various PROMs for upper and lower limbs without clear comparability.

## Figures and Tables

**Table 1:** Selection of validated PROMs for the assessment of Health-Related Quality of Life (HRQoL) in ULL patients and availability in German.

Validated PROMs for ULL	Available in German
Revised Lymphedema Functioning, Disability, and Health Questionnaire for Upper Limb (Lymph-ICF-UL) [21]	Yes [22]
Lymphedema Life Impact Scale (LLIS) [23]	No
Lymphedema Symptom Intensity and Distress Survey—Arm (LSIDS-A) [24]	No
LYMPH-Q Upper Extremity Module [25]	Yes [26]
Lymphedema Quality of Life Inventory (LyQLI) [27]	No
Quality of life scale in upper limb lymphedema (ULL-27) [28]	No
Upper Limb Lymphedema Quality of Life Questionnaire (ULLQoL) [29]	No
Freiburg Life Quality Assessment for Lymphedema (Short) (FLQA-L and FLQA-LS) [30,31]	Yes [30,31]
Lymphedema Quality of Life Questionnaire Arm (LYMQOL A) [32]	No

**Table 2:** Study population surveyed to translate and validate the German version of the LYMQOL Arm.

	LYMQOL Arm
Number of participants	52
Age	59.8 ± 10.8 (25–79)
Sex (male/female)	3/49; 5.7%/94.2%
Time between diagnosis and questioning	12.1 ± 10.1 (1–57) years
Body weight	81.0 ± 18.1 (54–135) kg
Height	167.4 ± 8.2 (150–188) cm
BMI	28.8 ± 5.7 (19–46) kg/m^2^
Employment	20 employed/2 self-employed/26 pensioned/2 unemployed/2 missing
Graduation	27 secondary school/10 high school/14 university/ 1 missing
Smoking	5 smokers/34 non-smokers/11 ex-smokers/2 missing
**ISL Lymphedema Stage**	
Stage I	11 (22.1%)
Stage II	26 (50.0%)
Stage III	15 (27.9%)
**Limb affected by lymphedema**	
Left arm	27 (52%)
Right arm	25 (48%)
Bilateral	0

**Table 3:** The most common scales in both questionnaires.

	LYMQOL Arm	
	First Interview	Second Interview
**Perceived Swelling** (Modus/Median)		
Left arm	2/2	2/2
Right arm	3/3	1/2
**LYMQOL Domain Scores** (Mean ± standard deviation)		
Function	2.0 ± 0.7	2.0 ± 0.7
Appearance	2.0 ± 0.8	2.0 ± 0.8
Symptoms	2.2 ± 0.6	2.1 ± 0.7
Mood	2.0 ± 0.7	2.01 ± 0.7
LYMQOL score	6/7	5/6
**SF-36** (Mean ± standard deviation)		
Physical function (PF)	62.1 ± 25.8	61.2 ± 25.3
Role physical (RP)	42.3 ± 42.3	40.3 ± 42.2
Role emotional (RE)	67.3 ± 41.0	69.3 ± 40.3
Vitality (VT)	44.0 ± 17.2	44.4 ± 20.0
Mental health (MH)	64.8 ± 19.4	65.3 ± 21.3
Social functioning (SF)	74.2 ± 26.5	72.4 ± 26.2
Bodily pain (BP)	59.5 ± 26.3	55.7 ± 26.6
General health (GH)	48.0 ± 21.6	47.6 ± 19.8
Physical component summary (PCS)	49.5 ± 9.1	50.0 ± 10.7
Mental component summary (MCS)	51.2 ± 10.6	50.0 ± 9.7

**Table 4:** Face and content validity of the German LYMQOL Arm version.

		Yes	No	No Answer
1.	Was the questionnaire easy to answer?	51 (98.1%)	1 (1.92%)	0
2.	Was the number of questions appropriate?	49 (94.2%)	2 (3.9%)	1 (1.9%)
3.	Were the questions clear?	47 (90.4%)	4 (7.7%)	1 (1.9%)
4.	Is there an important area of life in which lymphedema impacts your quality of life that is not included in the questionnaire?	6/(11.5%)	45 (86.5%)	1 (1.9%)
5.	Was a question unnecessary?	0	52 (98.1%)	1 (1.9%)
6.	Do you have any comments about this questionnaire?	15 (28.9%)	36 (69.2%)	1 (1.9%)

**Table 5:** Correlations between LYMQOL Arm and SF-36 to examine construct validity. r_s_: Pearson’s correlation coefficient.

SF 36	PCS				MCS			
	First Interview		Second Interview		First Interview		Second Interview	
LYMQOL	r_s_	*p*	r_s_	*p*	r_s_	*p*	r_s_	*p*
Function	−0.33	0.16	−0.63	≤0.01				
Symptoms	−0.50	≤0.01	−0.50	≤0.01				
Appearance					−0.48	≤0.01	−0.37	0.08
Mood					−0.63	≤0.01	−0.73	≤0.01

**Table 6:** Statistical relationship between the LYMQOL Arm score and the ISL lymphedema stage.

	I	II	III	Kruskal–Wallis Test	r_s_	*p*
Number of participants	12	28	12	P		
Function	1.8 ± 1.0	2.0 ± 0.7	2.3 ± 0.5	0.87	0.27	0.05
Appearance	1.7 ± 0.9	2.0 ± 0.8	2.3 ± 0.8	0.163	0.27	0.05
Symptoms	1.9 ± 0,8	2.2 ± 0.6	2.1 ± 0.3	0.08	0.29	0.05
Mood	2.1 ± 1.1	2.1 ± 0.6	1.8 ± 0.6	0.41	−0.06	0.64
Global Score	6.4 ± 2.0	5.7 ± 1.9	5.8 ± 1.2	0.63	−0.13	0.39

r_s_ = Spearman’s rank correlation coefficient.

**Table 7:** The reliability of the German LYMQOL Arm assessed through test–retest consistency.

	First Interview	Second Interview					
	M ± SD	M ± SD	ICC	*p*	Difference Means	SEM	SRD
Function	2.0 ± 0.7	2.0 ± 0.7	0.71	≤0.01	0.0	0.10	0.71
Appearance	2.0 ± 0.8	2.0 ± 0.8	0.66	≤0.01	0.0	0.12	0.87
Symptoms	2.2 ± 0.6	2.1 ± 0.7	0.71	≤0.01	0.1	0.10	0.71
Mood	2.0 ± 0.7	2.0 ± 0.7	0.73	≤0.01	0.0	0.11	0.77
Global score	5.4 ± 2.5	6.0 ± 1.8	0.68	≤0.01	0.6	0.26	1.80

M ± SD = mean ± standard deviation. ICC = intraclass correlation coefficient. SEM = standard error of the measurement. SRD = smallest real difference.

**Table 8:** Cronbach’s alpha coefficients for the German adaptation of the LYMQOL Arm.

	First Interview		Second Interview
	Number of Items	Cronbach’s Alpha Coefficient	Cronbach’s Alpha Coefficient
Function	10	0.89	0.89
Appearance	6	0.85	0.90
Symptoms	6	0.79	0.87
Mood	6	0.85	0.89

**Table 9:** The factors analysis.

	Question Numbers	Component			
		1	2	3	4
Function	1	0.193	**0.379**	**0.344**	0.262
	2	**0.395**	**0.383**	0.197	0.453
	3	**0.303**	**0.790**	0.062	0.094
	4	0.274	**0.824**	**0.304**	0.142
	5	0.221	**0.308**	0.240	0.267.
	6	−0.055	**0.658**	**0.308**	0.131
	7	0.142	**0.878**	0.085	0.134
	8	−0.041	**0.850**	0.133	0.132
	9	**0.409**	0.269	0.117	**0.379**
	10	0.149	0.262	**0.590**	0.227
Appearance	11	0.006	0.209	**0.908**	0.292
	12	0.085	0.230	0.287	**0.926**
	13	0.149	0.142	**0.89**	**0.824**
	14	0.152	0.253	**0.833**	0.227
	15	**0.472**	0.022	**0.430**	0.000
	16	**0.328**	0.080	**0.641**	0.027
Symptoms	17	**0.374**	0.273	0.271	0.144
	18	**0.632**	0.049	0.102	0.028
	19	**0.510**	−0.012	0.144	0.107
	20	**0.498**	0.094	**0.435**	0.350
	21	**0.375**	0.260	**0.557**	0.232
	22	**0.657**	0.179	0.070	0.266
Mood	23	**0.504**	0.251	0.113	0.089
	24	**0.794**	−0.090	−0.029	0.054
	25	**0.862**	0.024	0.196	0.065
	26	**0.689**	0.217	0.237	0.223
	27	**0.601**	0.274	0.134	−0.077
	28	**0.808**	0.273	0.215	0.202

Explained variance: 59.5%; factors loading >0.3 are bold numbers.

**Table 10:** Floor and ceiling effects across LYMQOL Arm domains in both questionnaires.

	First Interview	Second Interview	First Interview	Second Interview
	Lowest Result (N/%)	Lowest Result (N/%)	Highest Result (N/%)	Highest Result (N/%)
Function	1/1.9%	1/1.9%	7/13.4%	0/0%
Appearance	6/11.5%	5/9.6%	2/3.8%	3/5.7%
Symptoms	6/11.5%	1/1.9%	1/1.9%	1/1.9%
Mood	5/9.6%	5/9.6%	1/1.9%	1/1.9%

## Data Availability

The data presented in this study are available in the publicly accessible repository Zenodo at https://zenodo.org/records/12804259 (accessed on 24 July 2024).

## Supplementary Materials

The following supporting information can be downloaded at: https://www.mdpi.com/article/10.3390/healthcare12181881/s1, translated, cross-culturally adapted, and validated German version of Keeley et al.’s LYMQOL Arm [32] by Schulz et al.
