# Supplementary material for: Translation, Cross-Cultural Adaptation and Validation of the Lymphedema Quality of Life Questionnaire (LYMQOL) in German-Speaking Patients with Upper Limb Lymphedema
Source: Healthcare (Basel). 2024 Sep 20;12(18):1881. doi: 10.3390/healthcare12181881 (PMC11431024; doi:10.3390/healthcare12181881)
Supplement: Supplementary file 1 [file healthcare-12-01881-s001.zip › healthcare-3152314-supplementary.pdf]

Fragebogen zur Erfassung der Lebensqualität bei Lymphödem des Armes

LYMQOL Arm

Sehr geehrte Patient*innen,
der vorliegende Fragebogen dient der Erfassung des Einflusses des bei Ihnen bestehenden Lymphödems auf Ihre Lebensqualität. Bitte beziehen Sie dabei alle Fragen auf die Schwellung selbst, die notwendigen Behandlungen (bspw. manuelle Lymphdrainage, Kompressionskleidung) sowie eventuelle auftretende Komplikationen (bspw. Erysipel/Wundrose). Vielen Dank!

Alternativ: Patient*innenetikett

Name: ______________________________

Geburtsdatum: ______________________________

	Datum: ______________________	gar nicht (1)	etwas (2)	recht stark (3)	sehr stark (4)	n. z. nicht zutreffend
	**1. Als wie stark empfinden Sie die Schwellung des linken Armes?**	☐	☐	☐	☐	☐
	**2. Als wie stark empfinden Sie die Schwellung des rechten Armes?**	☐	☐	☐	☐	☐
FUNKTION	**3. Wie stark beeinträchtigen der geschwollene Arm und seine Behandlung Sie bei folgenden Alltagsaktivitäten?**					
	a. Beruf	☐	☐	☐	☐	☐
	b. Hausarbeit	☐	☐	☐	☐	☐
	c. Haare kämmen	☐	☐	☐	☐	☐
	d. Ankleiden	☐	☐	☐	☐	☐
	e. Schreiben	☐	☐	☐	☐	☐
	f. Essen	☐	☐	☐	☐	☐
	g. Waschen (Körperpflege)	☐	☐	☐	☐	☐
	h. Zähne putzen	☐	☐	☐	☐	☐
	**4. Wie stark sind Ihre Freizeitaktivitäten / Ihr Sozialleben dadurch beeinträchtigt?**	☐	☐	☐	☐	☐
	Bitte nennen Sie Beispiele:					
	**5. Wie sehr sind Sie auf Hilfe angewiesen?**	☐	☐	☐	☐	☐
KÖRPERBILD	**6. Wie sehr denken Sie, verändert die Schwellung Ihr Aussehen?**	☐	☐	☐	☐	☐
	**7. Wie schwierig ist es, Kleidung zu finden, die Ihnen passt?**	☐	☐	☐	☐	☐
	**8. Wie schwierig ist es, Kleidung zu finden, die Sie gerne tragen?**	☐	☐	☐	☐	☐
	**9. Beeinflusst die Schwellung Ihr Selbstbild?**	☐	☐	☐	☐	☐
	**10. Beeinflusst die Schwellung Ihre Partnerschaft?**	☐	☐	☐	☐	☐
	**11. Beeinflusst die Schwellung Ihre Beziehungen zu anderen Menschen?**	☐	☐	☐	☐	☐

BITTE WENDEN → Fortsetzung auf der Rückseite

		gar nicht (1)	etwas (2)	recht stark (3)	sehr stark (4)	n. z. nicht zutreffend
SYMPTOME	12. Haben Sie Schmerzen durch das Lymphödem?	☐	☐	☐	☐	☐
	13. Verspüren Sie ein Taubheitsgefühl im geschwollenen Arm?	☐	☐	☐	☐	☐
	14. Verspüren Sie ein Kribbeln oder „Ameisenlaufen“ im geschwollenen Arm?	☐	☐	☐	☐	☐
	15. Fühlt sich der geschwollene Arm schwach an?	☐	☐	☐	☐	☐
	16. Fühlt sich der geschwollene Arm schwer an?	☐	☐	☐	☐	☐
	17. Fühlen Sie sich müde oder erschöpft?	☐	☐	☐	☐	☐
STIMMUNG	**Hatten Sie in der vergangenen Woche…**					
	18. Probleme mit Schlaf?	☐	☐	☐	☐	☐
	19. Konzentrationsschwierigkeiten, z.B. beim Lesen?	☐	☐	☐	☐	☐
	20. Gefühle der Anspannung?	☐	☐	☐	☐	☐
	21. Sorgen?	☐	☐	☐	☐	☐
	22. Erhöhte Reizbarkeit?	☐	☐	☐	☐	☐
	23. Niedergeschlagenheit?	☐	☐	☐	☐	☐

24. Wie würden Sie Ihre Lebensqualität derzeit insgesamt bewerten?
Bitte vergeben Sie einen Punktwert auf der folgenden Skala:

☐	☐	☐	☐	☐	☐	☐	☐	☐	☐	☐
0	1	2	3	4	5	6	7	8	9	10

schlecht → hervorragend

Vielen Dank für Ihre Teilnahme!

Der vorliegende Fragebogen ist eine adaptierte Übersetzung des englischen LYMQOL Leg, welcher von Prof. Vaughan Keeley und Kollegen etabliert wurde (Keeley V, Crooks S, Locke J, et al. A quality of life measure for limb lymphoedema (LYMQOL). Journal of Lymphoedema 2010).

Bei Fragen oder Anmerkungen zu diesem Fragebogen wenden Sie sich bitte an:
Dr. med. Rima Nuwayhid
Klinik für Orthopädie, Unfallchirurgie und Plastische Chirurgie
Universitätsklinikum Leipzig AöR
Liebigstraße 20, 04103 Leipzig

Für die Untersuchenden: Auswertung
Pro Domäne werden die den Antworten zugeordneten Zahlenwerte addiert und durch die Anzahl der Fragen pro Domäne dividiert. Hierdurch ergeben sich Werte von 0 bis 4, wobei höhere Werte eine stärkere Einschränkung der Lebensqualität widerspiegeln. Sind die Hälfte oder mehr Fragen einer Domäne nicht beantwortet oder als nicht zutreffend gekennzeichnet, kann diese Domäne nicht ausgewertet werden.

	Funktion (÷ 10)	Körperbild (÷ 6)	Symptome (÷ 6)	Stimmung (÷ 6)
**ERGEBNIS**				
